# Small Incidental Pheochromocytoma Presenting With Normal or Borderline High 24-hour Urine Fractionated Metanephrines

**DOI:** 10.1210/jcemcr/luae035

**Published:** 2024-03-14

**Authors:** Kevin Jun Hong Kwek, Chin Pin Yeo, Bernard Chi Sern Ho, Yin Chian Kon

**Affiliations:** Department of Endocrinology, Tan Tock Seng Hospital, 308433, Singapore; Department of Clinical Pathology, Singapore General Hospital, 169608, Singapore; Department of Pathology, Tan Tock Seng Hospital, 308433, Singapore; Department of Endocrinology, Tan Tock Seng Hospital, 308433, Singapore

**Keywords:** pheochromocytoma, adrenal incidentaloma, plasma metanephrines

## Abstract

A 73-year-old man was found to have a 2-cm lipid-poor right adrenal incidentaloma on computed tomography imaging for hematuria. Twenty-four-hour urine metanephrine was 1.1-fold elevated, then normal on repeat measurement. Paired with the second urine collection, plasma metanephrine measured by liquid chromatography tandem mass spectrometry after a 30-minute supine rest was 3.3-fold elevated. Plasma normetanephrine was 1.2-fold elevated. The 24-hour urine catecholamines and normetanephrine, measured twice, were normal. He received low-dose phenoxybenzamine and underwent successful resection of right pheochromocytoma. Postoperatively, both plasma metanephrine and normetanephrine levels normalized, using an age-appropriate upper reference limit for plasma normetanephrine. Patients who harbor small lipid-poor adrenal incidentalomas have a relatively high risk (>5%) of having pheochromocytoma, indistinguishable from adenomas or carcinomas on computed tomography scan. In such cases when 24-hour urine fractionated metanephrines are normal, plasma free metanephrines measured by liquid chromatography tandem mass spectrometry under optimal sampling conditions that are 2-fold or more elevated confirm the diagnosis of pheochromocytoma. Preoperative alpha blockade followed by surgical resection is then appropriate, rather than continued monitoring with repeat urine measurements.

## Introduction

Pheochromocytoma (PCC) accounts for up to 7% of adrenal incidentalomas, defined as a clinically unapparent adrenal mass greater than 1 cm in diameter, detected during imaging for reasons other than suspected adrenal disease (1). Twenty to 60% of “modern day” PCCs present as adrenal incidentalomas (2). Small PCCs are challenging to diagnose because they are subclinical, computed tomography (CT) findings are nonspecific, and urine metanephrines normal or borderline elevated. Dismissing or delaying the diagnosis may lead to future cardiovascular complications. We present a case of small, lipid-poor adrenal incidentaloma with normal or equivocally elevated 24-hour urine metanephrines results. Subsequent paired plasma free metanephrine level was diagnostically elevated for PCC, leading to appropriate management.

## Case Presentation

A 73-year-old man was referred for evaluation of an incidentally discovered right adrenal nodule on abdominal CT imaging, performed for evaluation of hematuria. He had had hypertension for more than 10 years. On both losartan 50 mg and atenolol 25 mg once daily, his home blood pressure (BP) was 120/60 mm Hg to 140/80 mm Hg. In August 2021, the patient developed palpitations during cataract surgery under moderate sedation resulting from atrial flutter with variable atrioventricular conduction block. In November 2021, transthoracic echocardiogram revealed normal left ventricular ejection fraction (60%) without regional wall motion abnormalities, valvular dysfunction, or atrial dilation. He received rate control with atenolol and anticoagulation with apixaban. Coronary angiogram performed to investigate positive exercise stress electrocardiography revealed mild diffuse atherosclerotic disease. He had no previous history of thyroid, parathyroid, or renal tumors. There was no family history to suggest genetic pheochromocytoma or paraganglioma (PPGL). He had stopped smoking more than 10 years ago and consumed alcohol only socially. His BP measured 144/82 mm Hg, pulse was irregular with rate of 77 beats per minute. His weight was 67.3 kg, with a body mass index 23.8 kg/m^2^. He did not appear cushingoid. He was clinically euthyroid with no goiter. The cardiovascular and rest of the examinations were unremarkable.

## Diagnostic Assessment

**Figure 1.**
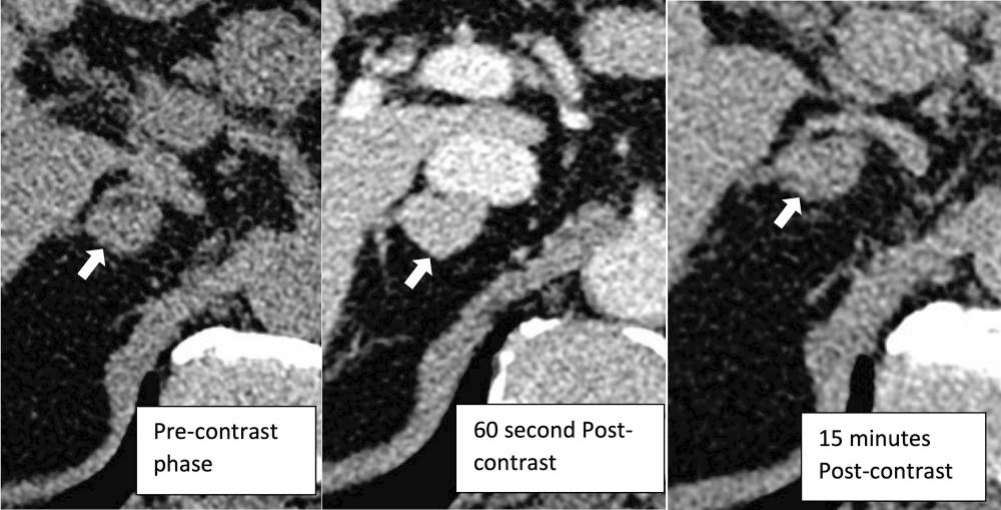
Contrast-enhanced CT adrenal showing a 2.0 cm × 1.3 cm homogenously enhancing right adrenal lesion (white arrow).

In April 2022, contrasted abdominal CT scan for hematuria revealed an indeterminate 1.9 × 1.3 cm right adrenal nodule. In June 2022, dedicated adrenal CT scan redemonstrated the 2.0 cm × 1.3 cm right adrenal nodule (Fig. 1). It appeared well circumscribed and homogenous without cystic degeneration, hemorrhage, or necrosis. The attenuation was 35 Hounsfield units (HU) precontrast, 102 HU at 60 seconds postcontrast, and 64 HU at 15 minutes postcontrast, giving an absolute washout of 56.7% and relative washout of 37.3%. The increased precontrast attenuation greater than 10 HU and delayed contrast washout (absolute washout < 60%, relative washout < 40%) were concerning for lipid-poor adrenal adenoma, PCC, or adrenal carcinoma. The left adrenal gland appeared normal.

Initial hormonal evaluation excluded Cushing syndrome and primary aldosteronism. However, 24-hour urine metanephrine (MN) was mildly elevated at 1.1 times the upper reference limit (URL). The 24-hour urine normetanephrine (NMN) level was normal (Table 1).

**Table 1.** Initial hormonal assessment

Hormone tested	Result	Reference range
8 Am cortisol	15.8 mcg/dL (435 nmol/L)	6.71-22.6 mcg/dL (185-624 nmol/L)
8 Am cortisol after overnight 1-mg dexamethasone suppression test	1.41 mcg/dL (39 nmol/L)	<1.81 mcg/dL (<50 nmol/L)
Aldosterone	<4.0 ng/dL (<111 pmol/L)	≤21 ng/dL (≤583 pmol/L)
Plasma renin activity	9.4 ng/mL/h (222.8 pmol/L/h)	.6-4.3 ng/mL/h (14.2-102 pmol/L/h) Assuming Na-replete, upright
24-h urine noradrenaline	61 mcg/d (362 nmol/d)	15-80 mcg/d (89-473 nmol/d)
24-h urine adrenaline	11 mcg/d (59 nmol/d)	.5-20 mcg/d (3-109 nmol/d)
24-h urine normetanephrine	330 mcg/d (1803 nmol/d)	162-528 mcg/d (885-2880 nmol/d)
24-h urine metanephrine	**340 mcg/d** **(1725 nmol/d)**	64-302 mcg/d (325-1530 nmol/d)
24-h urine dopamine	131 mcg/d (855 nmol/d)	65-400 mcg/d (424-2612 nmol/d)

Twenty-four-h urine collection volume for metanephrines and catecholamines from December 3 to 4, 2022, was 878 mL. Abnormally high values are shown in bold font. Values in parentheses are International System of Units.

With this background history, we were concerned not to miss a diagnosis of PCC, given the slightly raised 24-hour urine MN and CT imaging characteristics of the adrenal nodule (1, 2). In cases of borderline increases of urinary fractionated metanephrines, follow-up measurements with plasma metanephrines are appropriate (3). Thus, we repeated 24-hour urine fractionated metanephrines paired with plasma-free metanephrines (liquid chromatography tandem mass spectrometry [LC-MS/MS]), obtained after an overnight fast and supine rest in a quiet room for 30 minutes. He was not taking any sympathomimetic agents, caffeine, recreational drugs, or interfering medications such as tricyclic antidepressants, monoamine oxidase inhibitors, or L-Dopa before testing. His repeat 24-hour urine MN and NMN levels were normal. However, plasma MN level was 3.3-fold elevated and plasma NMN level 1.2-fold elevated. His 24-hour urine catecholamine levels were normal on initial and repeat testing (Table 2). The elevated plasma MN level at more than 3 times the URL confirmed the diagnosis of adrenergic predominant PCC (4, 5). We advised early adrenalectomy while the MN excess was relatively mild and PCC was small, making surgery safer (6).

**Table 2.** Second preoperative hormonal assessment

Hormone tested	Result	Reference range
24-h urine noradrenaline	26 mcg/d (151 nmol/d)	15-80 mcg/d (89-473 nmol/d)
24-h urine adrenaline	8.2 mcg/d (45 nmol/d)	.5-20 mcg/d (3-109 nmol/d)
24-h urine normetanephrine	152 mcg/d (835 nmol/d)	162-528 mcg/d (885-2880 nmol/d)
24-h urine metanephrine	256 mcg/d (1297 nmol/d)	64-302 mcg/d (325-1530 nmol/d)
24-h urine dopamine	77 mcg/d (501 nmol/d)	65-400 mcg/d (424-2612 nmol/d)
Plasma metanephrine	**.22 mcg/L** **(1.08 nmol/L)**	<.07 mcg/L (<.33 nmol/L)
Plasma normetanephrine	**.21 mcg/L** **(1.20 nmol/L)**	<.14 mcg/L URL for age 50-60 y <.18 mcg/L URL for age >60 y (<.80 nmol/L URL for age 50-60 y) (<.99 nmol/L URL for age >60 y)

Repeat 24-h urine collection volume for catecholamines and metanephrines from December 19 to 20, 2022, was 1864 mL. Plasma metanephrines were sampled on December 20, 2022, and measured at the Clinical Biochemistry Laboratory, Singapore General Hospital, using Agilent 6460 triple quadrupole liquid chromatography-tandem mass spectrometer and Recipe ClinMass Free Metanephrines assay kit. Within-run imprecision coefficients of variation (CVs) for both plasma metanephrines and normetanephrines assays, using manufacturer's quality control materials, were below 6%, whereas the overall total imprecision CVs of both assays were less than 7%. Abnormally high values are shown in bold font. Values in parenthesis are International System of Units.

Abbreviation: URL, upper reference limit.

## Treatment

He was started on oral phenoxybenzamine 10 mg once daily for preoperative alpha blockade. Oral losartan was discontinued. Oral atenolol 25 mg every morning was continued for rate control of his atrial arrhythmia. Although beta blockade should be avoided without concurrent alpha blockade in patients who harbor PPGL, we noted that his BP was stably controlled before initiation of alpha blockade. Subsequent home BP readings ranged from 100 to 130/60 to 80 mm Hg and heart rate 50 to 70 beats per minute, without postural giddiness or orthostatic hypotension. Phenoxybenzamine was ceased 24 hours before laparoscopic adrenalectomy, intravenous 2 L/day .9% sodium chloride infusion started, and oral atenolol continued.

**Figure 2.**
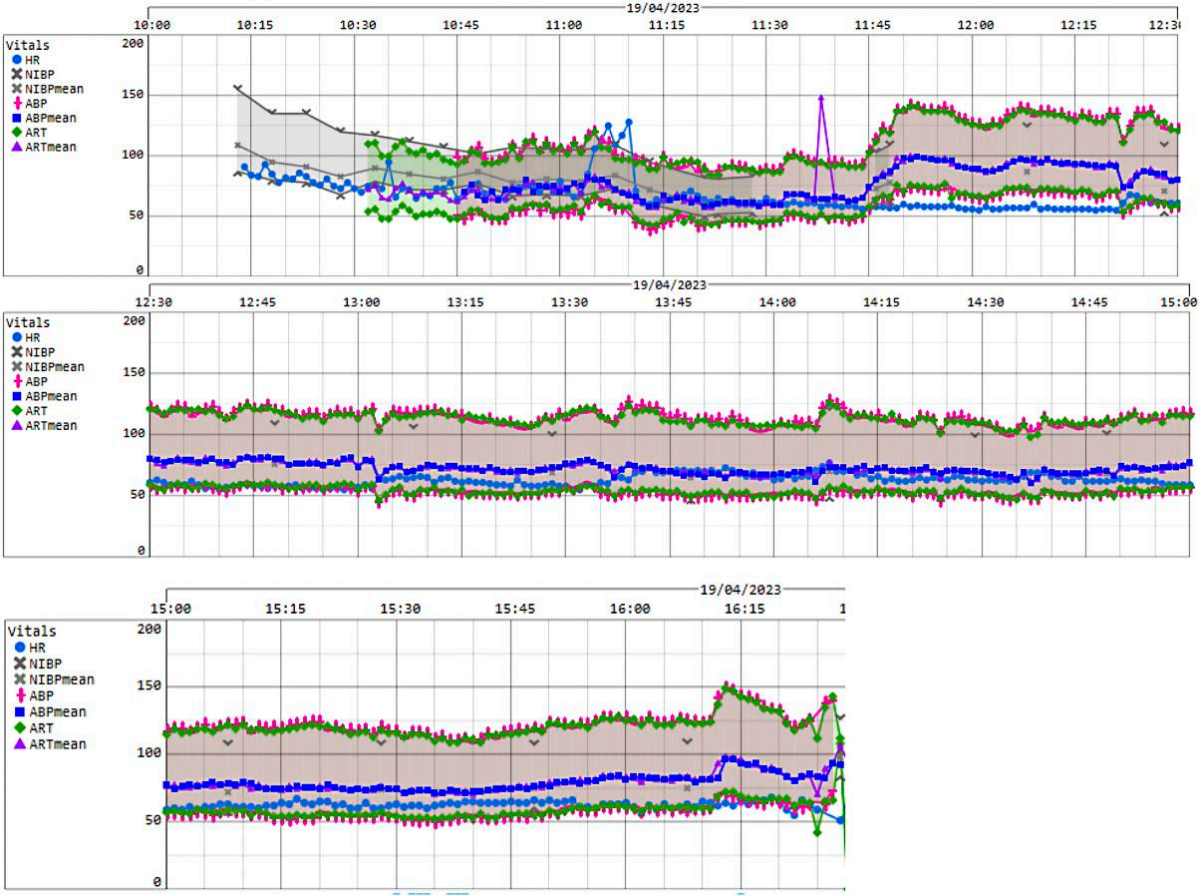
Intraoperative blood pressure and heart rate charts.

After anesthesia induction with IV 1 mg of midazolam and IV 70 mg of rocuronium, our patient experienced transient hypotension with nadir systolic blood pressure of 90 mm Hg, requiring 6 doses of IV phenylephrine totaling 300 mcg (Fig. 2). A brief 60-second episode of atrial fibrillation with rapid ventricular rate occurred after phenylephrine administration, which resolved without any IV beta blockers. Anesthesia was maintained with remifentanil and propofol infusions. Subsequent intraoperative hemodynamic stability was maintained on low-dose norepinephrine infusion at .01 mcg/kg/min and IV magnesium sulfate infusion at 4 mmol/h. Intraoperative capillary blood glucose ranged from 6 to 8 mmol/L.

The entire right adrenal gland containing the 2.0 × 1.8 × 1.2 cm well-circumscribed PCC was removed intact without rupture of the capsule. Unfortunately, the surgery was complicated by inferior vena cava injury requiring conversion to open surgery for repair. Subsequent postoperative course was uneventful, his blood pressure measured 125 to 146/60 to 80 mm Hg with a heart rate of 55 to 80/min, with normal capillary blood glucose readings. Oral losartan 50 mg daily was resumed on discharge on the afternoon of the second postoperative day.

## Outcome and Follow-up

**Figure 3.**
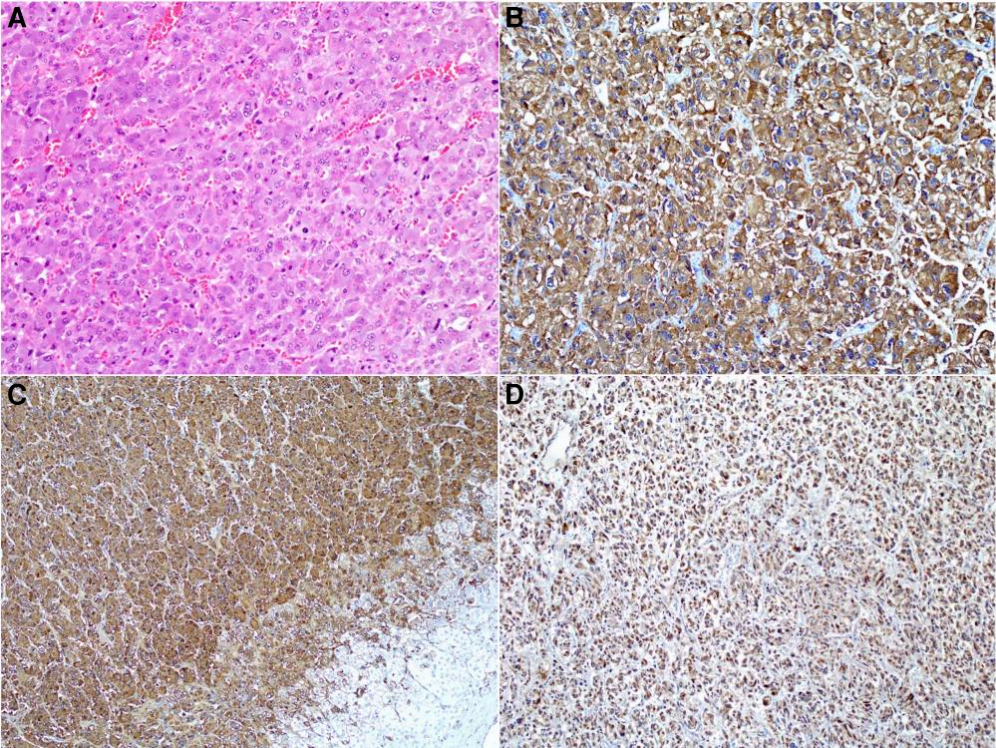
(A) The adrenal tumor composed of compact nests of polygonal cells with abundant purplish granular cytoplasm and round to oval nuclei with small nucleoli (hematoxylin and eosin ×100). (B) Tumor cells stain positive for synaptophysin (×200). (C) Tumor cells also stained positive for chromogranin. Note that the normal adrenal cortical cells are negative for this stain (bottom right) (×100). (D) Tumor cells retain cytoplasmic SDHB staining, implying absence of SDHx germline mutations (×200).

Gross pathology confirmed the diagnosis of PCC. On microscopy, nests of polygonal cells staining positive for synaptophysin and chromogranin on immunohistochemistry (IHC) were seen (Fig. 3). No areas of necrosis or brisk mitotic activity were seen within the lesion. Clear resection margins were seen with no extra-adrenal extension nor involvement of lymph nodes. IHC staining for succinate dehydrogenase B (SDHB) was positive, which suggested absence of underlying germline succinate dehydrogenase X (SDHx) mutations (7, 8).

At outpatient review, our patient remained well with normal BP at 110 to 130/70 to 90 mm Hg on atenolol 25 mg and losartan 50 mg daily. His plasma MN had normalized; however, plasma NMN remained slightly elevated on 1 of 2 different LC-MS/MS laboratory platforms (Table 3). Because metanephrine excess is proportionate to tumor bulk, he was unlikely to have had distant metastatic disease or extra-adrenal disease at presentation (9, 10). Histological and intraoperative reports had both confirmed complete resection of the PCC. Normal positive IHC staining for SDHB made presence of small paraganglioma resulting from germline SDHx mutation unlikely. Hence, we concluded that our patient had been surgically cured and attributed the “mildly elevated” postoperative plasma NMN to an inappropriately low URL for his age (11, 12). To be prudent, his plasma and/or 24-hour urine metanephrines will be monitored on a yearly basis to detect recurrence.

**Table 3.** Postoperative hormonal assessment

Hormone tested	Result	Reference range
Postoperative day 40		
24-h urine noradrenaline	32 mcg/d (190 nmol/d)	15-80 mcg/d (89-473 nmol/d)
24-h urine adrenaline	7.3 mcg/d (20 nmol/d)	.5-20 mcg/d (3-109 nmol/d)
24-h urine normetanephrine	158 mcg/d (862 nmol/d)	162-528 mcg/d (885-2880 nmol/d)
24-h urine metanephrine	248 mcg/d (1258 nmol/d)	64-302 mcg/d (325-1530 nmol/d)
24-h urine dopamine	129 mcg/d (843 nmol/d)	65-400 mcg/d (424-2612 nmol/d)
Plasma metanephrine***^a^***	.03 mcg/L (.16 nmol/L)	<.07 mcg/L (<.33 nmol/L)
Plasma normetanephrine***^a^***	.14 mcg/L (.81 nmol/L)	<.14 mcg/L URL for age 50-60 y <.18 mcg/L URL for age > 60 y (<.80 nmol/L URL for age 50-60 y) (<.99 nmol/L URL for age > 60 y)
Postoperative day 126		
Plasma metanephrine***^b^***	<.04 mcg/L (<.20 nmol/L)	<.1 mcg/L (<.50 nmol/L)
Plasma normetanephrine***^b^***	**.22 mcg/L** **(1.20 nmol/L)**	<.16 mcg/L (<.90 nmol/L)

Postoperative day forty 24-h urine collection volume was 1459 mL.

Abbreviation: URL, upper reference limit.

^*a*^Plasma metanephrines measurements were performed at the Clinical Biochemistry Laboratory, Singapore General Hospital, using Agilent 6460 triple quadrupole liquid chromatography-tandem mass spectrometer and Recipe ClinMass Free Metanephrines assay kit. Within-run imprecision coefficients of variation (CVs) for both plasma metanephrines and normetanephrines assays, using manufacturer's quality control materials, were below 6%, whereas the overall total imprecision CVs of both assays were less than 7%.

^*b*^Plasma metanephrines measurements were performed at Mayo Clinic Laboratories. Free metanephrine and normetanephrine are extracted from plasma using solid phase extraction. The concentrated eluate is analyzed using liquid chromatography tandem mass spectrometry and quantified using stable isotope-labeled internal standards, d3-metanephrine, and d3-normetanephrine. Bold font values are “abnormally high” as upper reference limit quoted by laboratory is unadjusted for age. Values in parenthesis are International System of Units (SI).

## Discussion

Although rare, PPGLs are potentially lethal and should not be missed. A highly sensitive test with high negative predictive value should be used to rule out the diagnosis (4, 5, 13-15). However, when disease prevalence is low, a highly sensitive test also confers lower specificity with a higher rate of false positives. The prevalence of PPGL is less than 1% to 2% in those who present only with suggestive symptoms and hypertension but is enriched to exceed 5% to 10% in those who present with hereditary risk of PPGL (40%), history of PPGL (16.5%), or as adrenal incidentalomas (4%-9%), especially in those with suspicious CT characteristics (2, 4). Confirming diagnosis of PPGL using a highly specific test for high positive predictive value is important because it mandates adequate preoperative preparation to avoid intraoperative catecholamine crisis. The posttest probability of PPGL increases when (1) pretest prevalence is relatively high, such as with suspicious looking adrenal incidentalomas, (2) a highly specific test is used, and (3) test result is many-fold higher than URL (4, 5, 13-15).

In a seminal prospective European multicenter study by Eisenhofer et al, 2056 patients with suspected PPGL were screened using LC-MS/MS-based measurements of plasma free, urinary free, and urinary deconjugated (ie, combined free plus sulfate-conjugated) metanephrines (4). PPGL was confirmed in 236 patients, giving a prevalence of 11.5%. Plasma free metanephrines were found to offer better diagnostic performance than 24-hour urinary metanephrines for patients at high risk of having PPGL, defined as those with (1) adrenal incidentaloma, (2) hereditary risk of PPGL, or (3) history of PPGL (4). In higher risk patients, when plasma free metanephrines (LC-MS/MS) after quiet supine rest measured more than 2 times the URL, the diagnostic specificity and positive predictive value for PPGL was 100% (4). In this group, plasma metanephrines were more sensitive than urinary free metanephrines: sensitivity 96.7% vs 89.6% respectively. Specificities (both at 92.8%), and area under the curve (.971; 95% CI, .949-.983 vs .958; 95% CI, .930-.975) were similar.

For patients at lower risk (ie, those tested because of signs and symptoms of presumed catecholamine excess), urinary and plasma tests display similarly high diagnostic performance (4, 5, 13-15). Measured by LC-MS/MS under correct sampling conditions, increases of both plasma NMN and MN are rare as false-positive results, and occur in at least half of all patients with PCC. Similarly, solitary increases in either plasma metabolite more than 2 times the URL, in particular in plasma MN, which is specific for adrenal origin, such as in our case, are also rare as false positives. Increase in plasma free NMN greater than 2.2 nM or plasma free MN greater than 1.2 nM is 3.5- to 4-fold above adult URL, such results make the presence of PPGL extremely likely (∼100% specificity) (14). With such magnitudes of elevation, the posttest probability of PPGL can be higher than 90%, even at pretest prevalence of only 1% (4, 5). Assuming pretest probability of PCC in our patient was 5% to 10%, we were confident our patient had PCC, given that his plasma MN was more than 3 times above the URL. When there is any doubt about PPGL diagnosis because plasma or 24-hour urine fractionated metanephrines are only marginally elevated because of small “factory” mass, ^123^I-metaiodobenzylguanidine or ^68^Ga-DOTATATE positron emission tomography scans that show avid uptake in the lesion can be useful diagnostic adjuncts (4, 16, 17). For patients with past PPGL or genetic risk, prolonged surveillance for up-trending results is essential (4, 18).

It has been shown that the URL for plasma free NMN increases with age, and age-group specific reference intervals improve diagnostic specificity while maintaining sensitivity (11, 12). One study using liquid chromatography-electrochemical detection suggested plasma NMN URL (97th percentile) to be .62 nmol/L for adults younger than age 40 years, and 1.05 nmol/L for adults older than age 60 years, to reflect the curvilinear rise seen on modeling the effect of age. No effect of age was found for plasma MN with URL at .45 nM (11). Applying the age-group-specific URL for plasma NMN measured by LC-MS/MS from our local laboratory (12), our 73-year-old patient was surgically cured (Table 3). His antihypertensive medications were continued to treat what was likely to be chronic essential hypertension.

## Learning Points

- Patients with primary small (≤2 cm), lipid-poor adrenal incidentalomas have a relatively high risk (>5%) of having PCC. The differentials are adenoma and adrenal carcinoma. The 24-hour urine metanephrines may be normal or borderline elevated.
- In such patients, plasma free metanephrines measured by LC-MS/MS, after a 30-minute supine rest, outperform 24-hour urine metanephrines and should be the screening test of choice. When sampling conditions are optimized, plasma free metanephrines more than 2 times the URL confirm the diagnosis of PCC and mandate surgical resection, rather than watchful waiting.
- The URL for plasma NMN has to be age-group specific and adjusted upwards accordingly to avoid false-positive interpretation in those older than age 60 years.

## Contributors

All authors made individual contributions to authorship. K.J.H.K. and Y.C.K. were involved in the diagnosis and management of the case. C.P.Y. and B.C.S.H. participated in the preparation of parts of manuscript. All authors reviewed and approved the final draft.

## Data Availability

Original data generated and analyzed for this case report are included in this published article.
